# Supplementary figures and images for: Phenotypic tolerance for rDNA copy number variation within the natural range of C. elegans
Source: PLoS Genet. 2025 Jul 2;21(7):e1011759. doi: 10.1371/journal.pgen.1011759 (PMC12221044; doi:10.1371/journal.pgen.1011759)

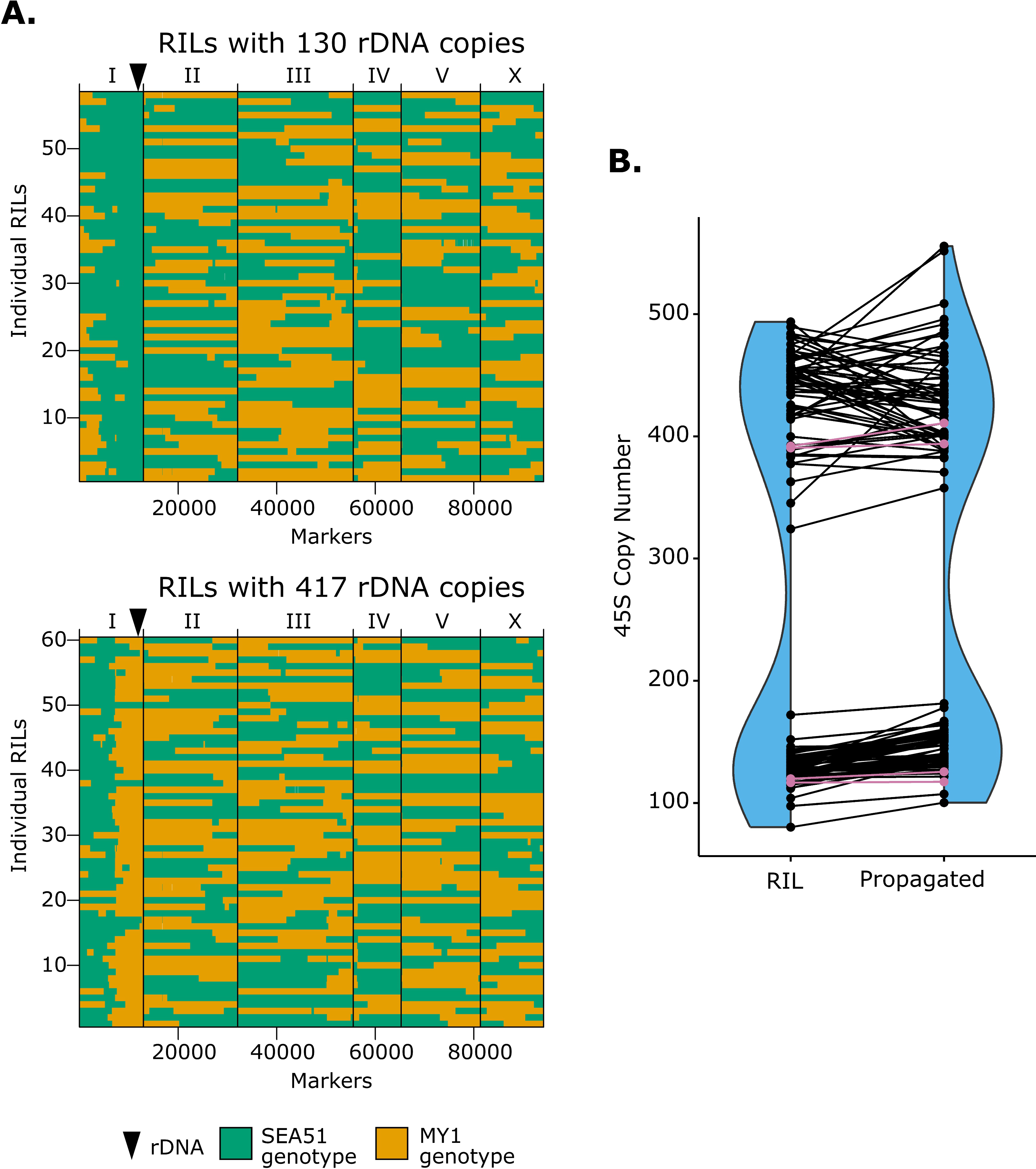

Supplement: S1 Fig — A: Top: The genotypes of the 58 RILs with ~130 rDNA copies are presented, with yellow representing haploblocks matching the parental MY1 wild isolate genotype and green representing the parental SEA51 N2-derivative genotype. Bottom: The genotypes of the 60 RILs with ~417 rDNA copies are presented. The black carat indicates the rDNA locus at the end of chromosome I. Genotypes were determined with GATK HaplotypeCaller and the map was filled with the max marginal method. B: Short-read sequencing-based 45S rDNA copy number estimates are given for RILs pre- and post-propagation for 20 generations. Data are the same as presented in Fig 1C and D but plotted here on a continuous axis. Pink lines indicate parental control strains (see Fig 1). (TIF) [file pgen.1011759.s001.tif]

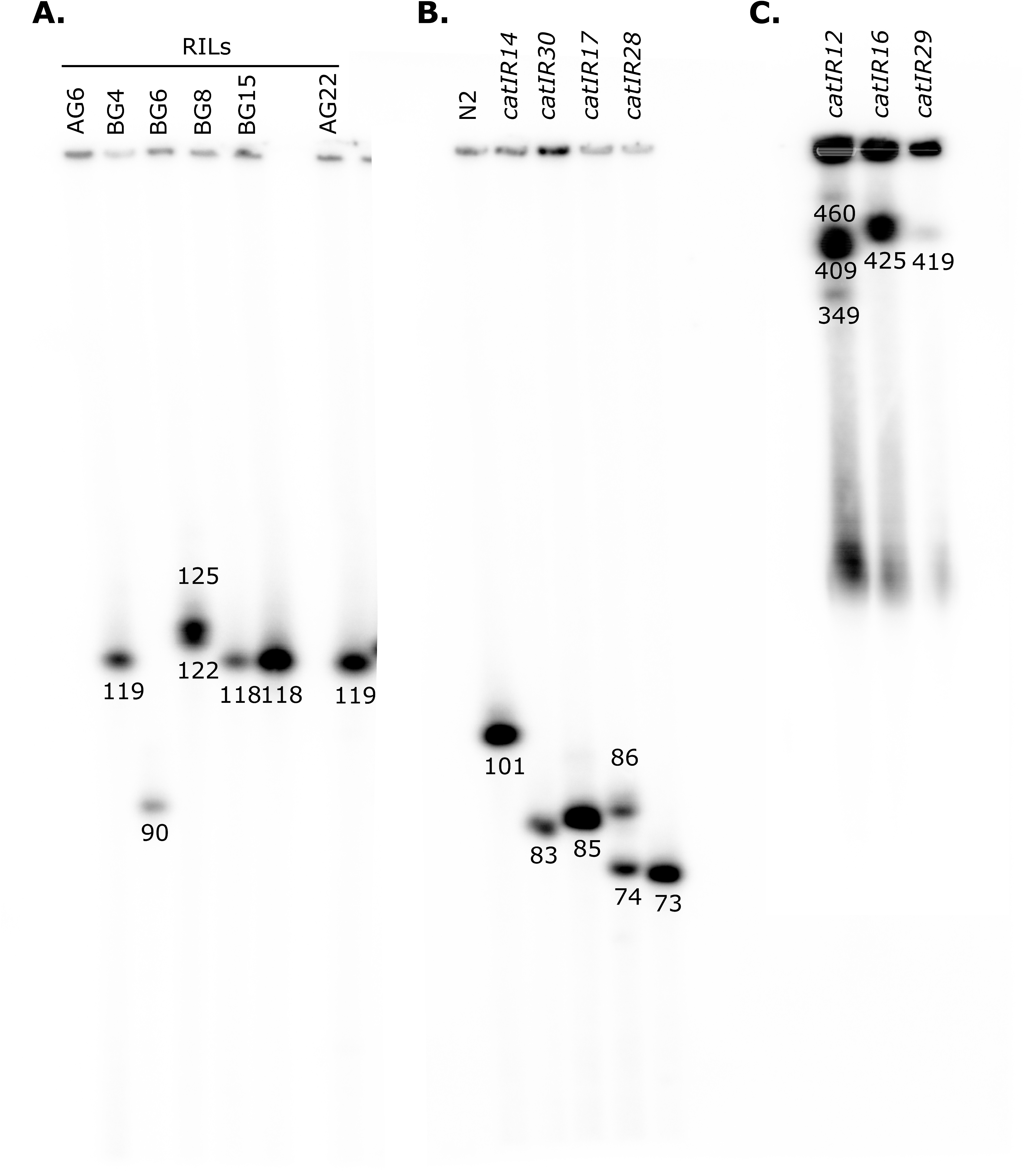

Supplement: S2 Fig — A: Six RILs with rDNA linked to the mIs13 transgene were analyzed by CHEF gel and Southern blot under conditions to resolve rDNA arrays near 100 copies in length (see S1 Table, Methods). Band sizes were calculated based on distance of band migration from the well compared to an S. cerevisiae chromosome ladder that was visualized by ethidium bromide staining prior to Southern blotting (S10 File). Copy numbers were calculated from the base pair size of the band divided by 7.2kb, the size of a single C. elegans rDNA unit. B: N2 and NIL rDNA arrays were separated with conditions that resolve arrays near 100 rDNA copies. Copy numbers were calculated as in A. N2 is in lane 1 and allele identifiers for introgressed rDNA arrays in NILs are indicated in subsequent lanes. C: NILs with high rDNA copy number were analyzed with CHEF gel conditions that resolve arrays larger than 200 copies. Copy numbers were calculated based on reference to an H. wingei chromosome ladder that was visualized by ethidium bromide staining prior to Southern blotting. Allele identifiers for rDNA arrays are indicated. Numbers on the blots indicate the calculated rDNA copy number for each band. (TIF) [file pgen.1011759.s002.tif]

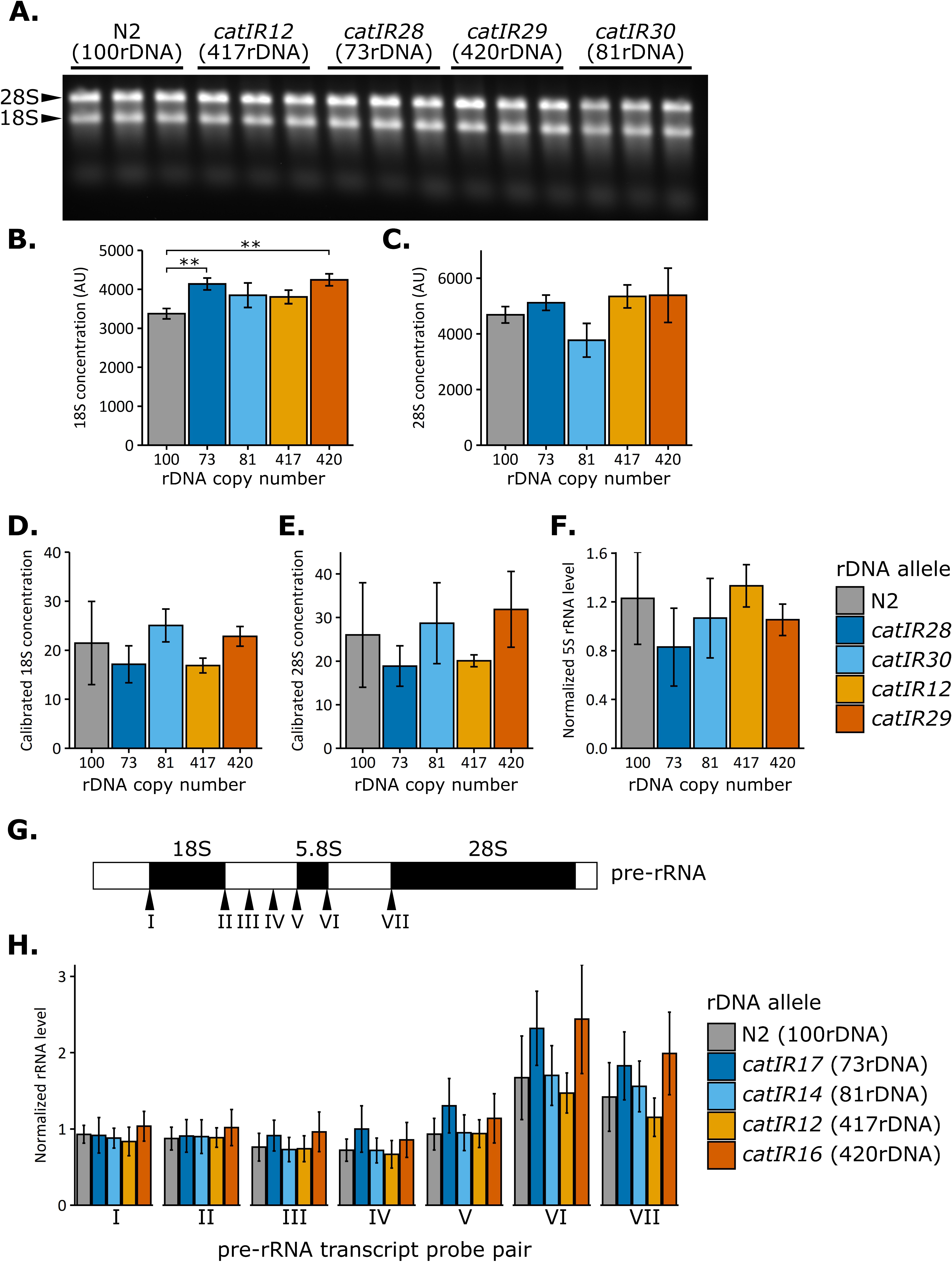

Supplement: S3 Fig — A: Equal quantities of RNA extracted from Day 1 adult worms were denatured and run on an agarose gel for each sample. Each lane is a separate biological replicate. B and C: Intensities of 28S and 18S bands from A were quantified with ImageJ. **: p < 0.01 as determined by ANOVA and Tukey’s HSD. D-F: Steady-state rRNA levels in NILs. D: 18S rRNA levels in NILs were measured by TapeStation. No significant differences in 18S rRNA levels were observed between any strains as assessed by ANOVA and Tukey’s HSD. E: 28S rRNA levels in NILs were measured by TapeStation. Data are not normally distributed as determined by the Shapiro-Wilk normality test. No significant differences in 28S rRNA levels were observed between any strains as measured by pairwise Wilcoxon test and Benjamini-Hochberg significance adjustment. F: 5S rRNA levels in NILs were measured by RT-qPCR, normalized to actin. No significant differences in 5S rRNA levels were observed between any strains as measured by ANOVA and Tukey’s HSD. Legend at the right indicates the rDNA allele for each strain in D-F. G: Diagram of 45S pre-rRNA processing in C. elegans, adapted based on Wu et al. 2018 [138]. Primer pairs are as indicated in S7 Table. H: Pre-rRNA levels were quantified by RT-qPCR in NILs with large regions of linked wild isolate DNA (see Fig 2A). rRNA levels are normalized to actin. Error bars are mean ± standard deviation. No significant differences are present between any NILs and N2 (ANOVA with Tukey Honest Significant Difference test). (TIF) [file pgen.1011759.s003.tif]

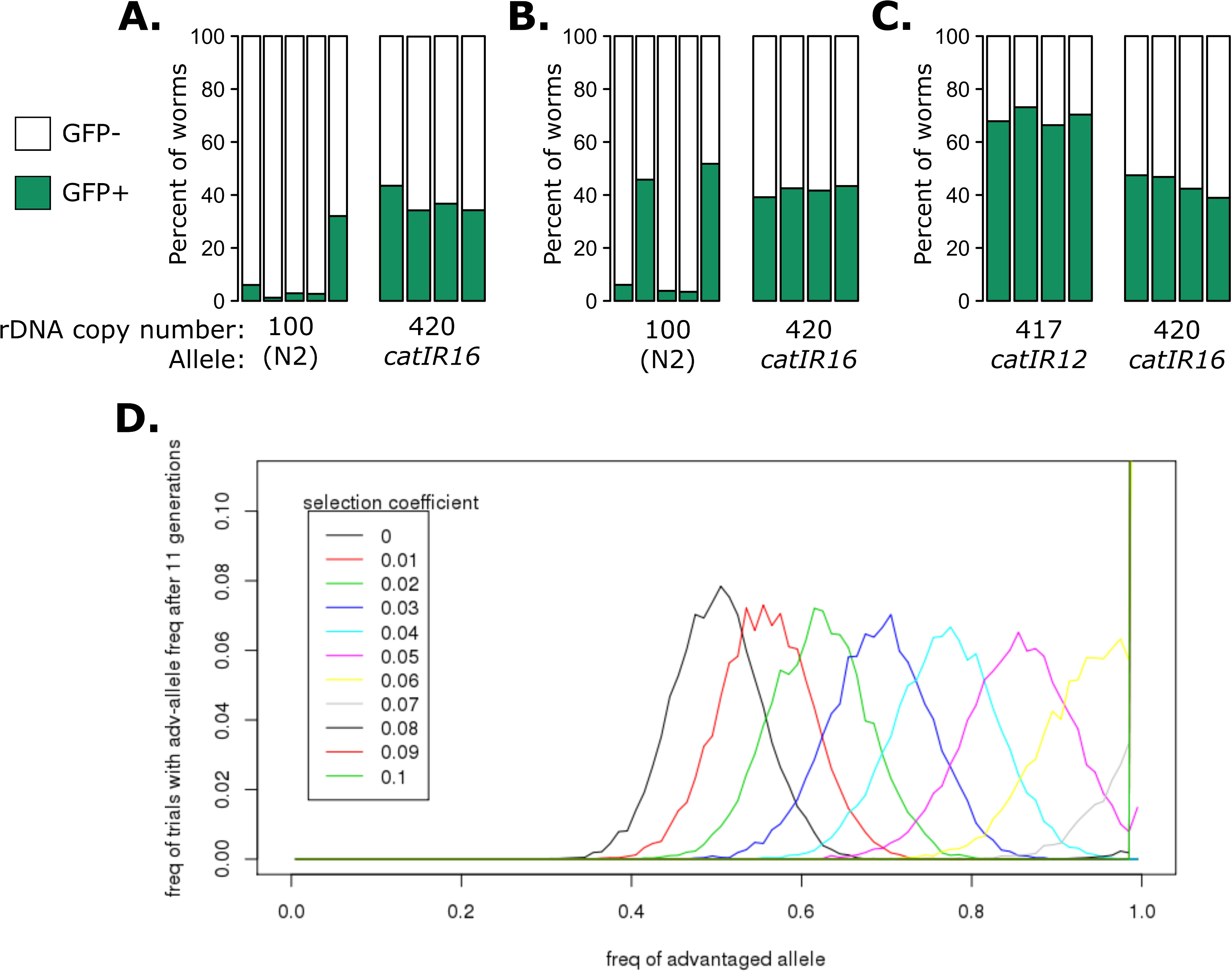

Supplement: S4 Fig — Competitions of strains with high rDNA copy number (GFP(-); alleles indicated at bottom) set against SEA51 (GFP(+); 130 rDNA copies). For panels A-C, each bar shows the relative proportion of worms that are GFP(+) (green) or GFP(-) (white) after ~10–11 generations of competition. At least 1,000 worms were quantified to determine the proportion of each bar (S14 File). A: Five independent replicates were conducted of SEA51 competed against N2, performed at the same time as four replicates of SEA51 competed against the 420-rDNA NIL (allele catIR16, which has ~ 3.3Mb of wild isolate DNA linked to the rDNA array (Fig 2A)). B: Experiment set up similarly to A. Five replicate competitions between SEA51 and N2 were performed, with four replicate competitions between SEA51 and 420-rDNA NIL (allele catIR16) performed at the same time. C: Competitions between SEA51 and each of the two strains with high rDNA copy number were performed side-by-side, with four independent replicates each. High rDNA copy number strains are the 417-rDNA NIL (allele catIR12) and 420-rDNA NIL (allele catIR16). D: A simulation of selection coefficients was used to determine what strength of selection is required to give a certain population proportion after 11 generations of propagation with a propagated population size of 1000. (TIF) [file pgen.1011759.s004.tif]

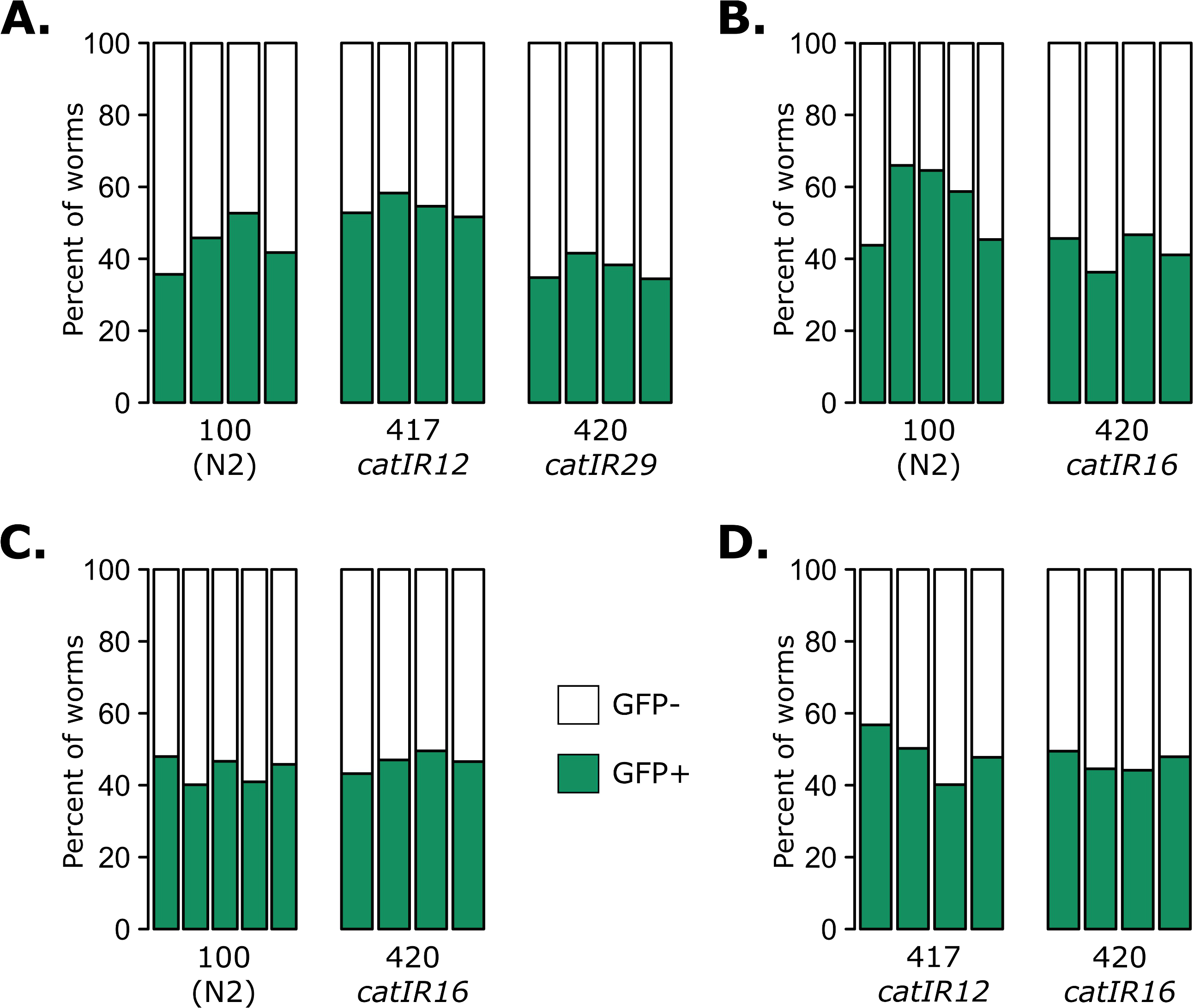

Supplement: S5 Fig — Competition population proportion data was collected early in the assay, corresponding to the point when the populations were transferred for the first time (out of eight total transfers in the assay), approximately 2–3 generations into the assay. A: Early timepoint data for the competitions presented in Fig 3B. Four plate replicates each are shown for SEA51 competing against either N2, the 417-rDNA NIL (allele catIR12), or the 420-rDNA NIL (allele catIR29). These three sets of competing pairs were assayed simultaneously. B: Early timepoint data for the competitions presented in S4A Fig. Data are shown for SEA51 competing against either N2 (five replicates) or the 420-rDNA NIL (allele catIR16) (four replicates), propagated at the same time. C: Early timepoint data for the competitions presented in S4B Fig. Data are shown for SEA51 competing against either N2 (five replicates) or the 420-rDNA NIL (allele catIR16) (four replicates), propagated at the same time. D: Early timepoint data for the competitions presented in S4C Fig. Competitions against SEA51 are shown for two strains with high rDNA copy number (417-rDNA NIL (allele catIR12) and 420-rDNA NIL (allele catIR16)), propagated at the same time. For all panels, each bar shows the relative proportion of worms that are GFP(+) (green) or GFP(-) (white) after ~10–11 generations of growth. At least 1,000 worms were quantified to determine the proportion of each bar (S14 File). (TIF) [file pgen.1011759.s005.tif]

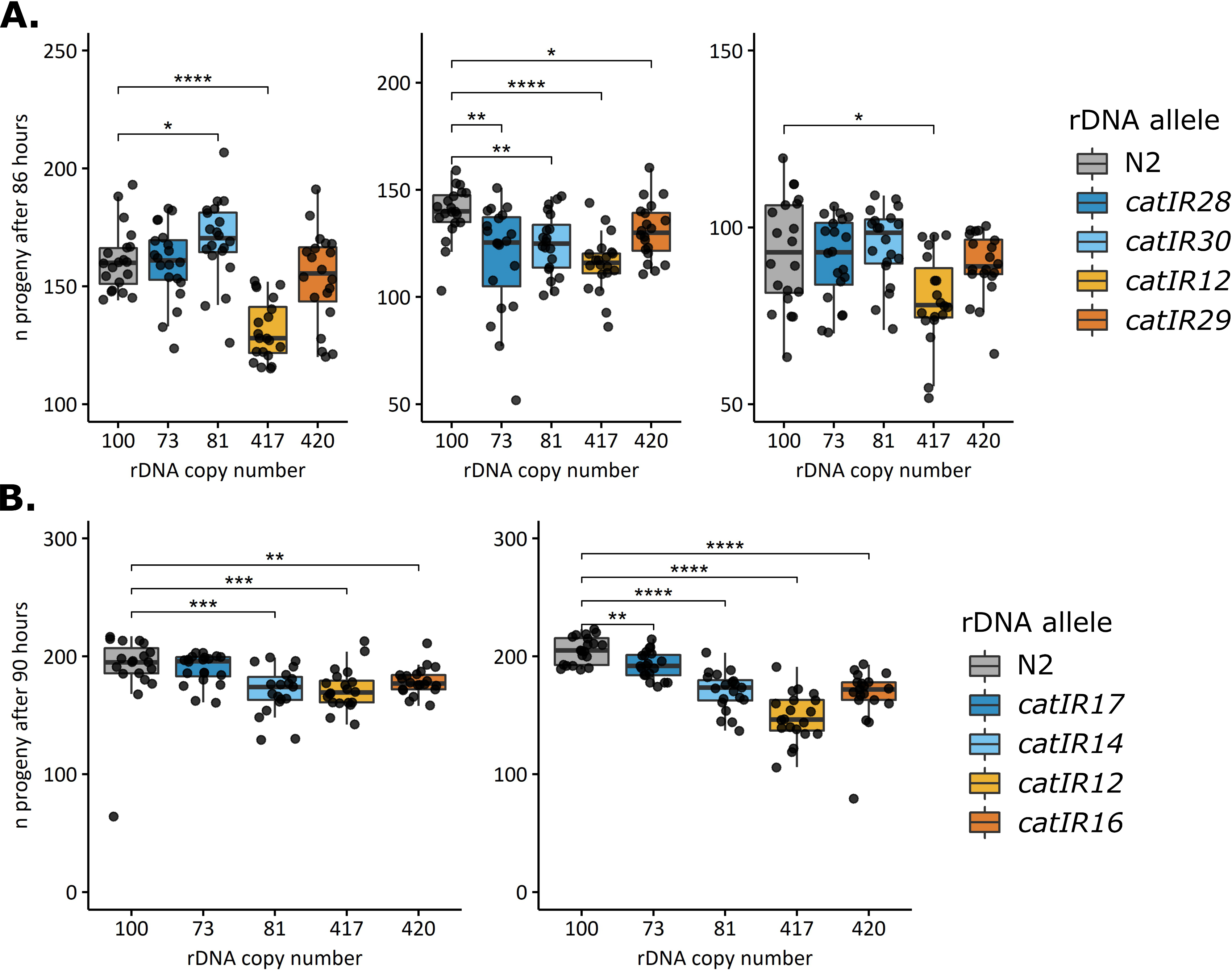

Supplement: S6 Fig — A: Early life fertility of NILs was compared to N2 in three replicates, n = 20 individual worms per strain per replicate. These strains represent the panel of NILs with minimal rDNA-linked wild isolate DNA. The data fail the Shapiro-Wilk normality test and are not normally distributed. A nonparametric Scheirer Ray Hare test of Progeny by Strain and Replicate shows a significant effect of Strain (p = 0.00137) and Replicate (p < 0.00001) across the three replicates. Due to the lack of normality and the significant effect of replicate, strain-by-strain comparisons were performed separately for each replicate. The data for N2, catIR12, and catIR29 are the same as the data presented in Fig 3C. B: Two replicates of the early life fertility assay were performed on the panel of NILs with large regions of rDNA-linked wild isolate DNA, n = 20 individual worms per strain per replicate. The data fail the Shapiro-Wilk normality test and are not normally distributed. For both panels A and B, statistical tests represented in the figure are Pairwise Wilcoxon tests with Benjamini-Hochberg procedure performed to compare strains. * p < 0.1, ** p < 0.05, *** p < 0.01, **** p < 0.001. (TIF) [file pgen.1011759.s006.tif]

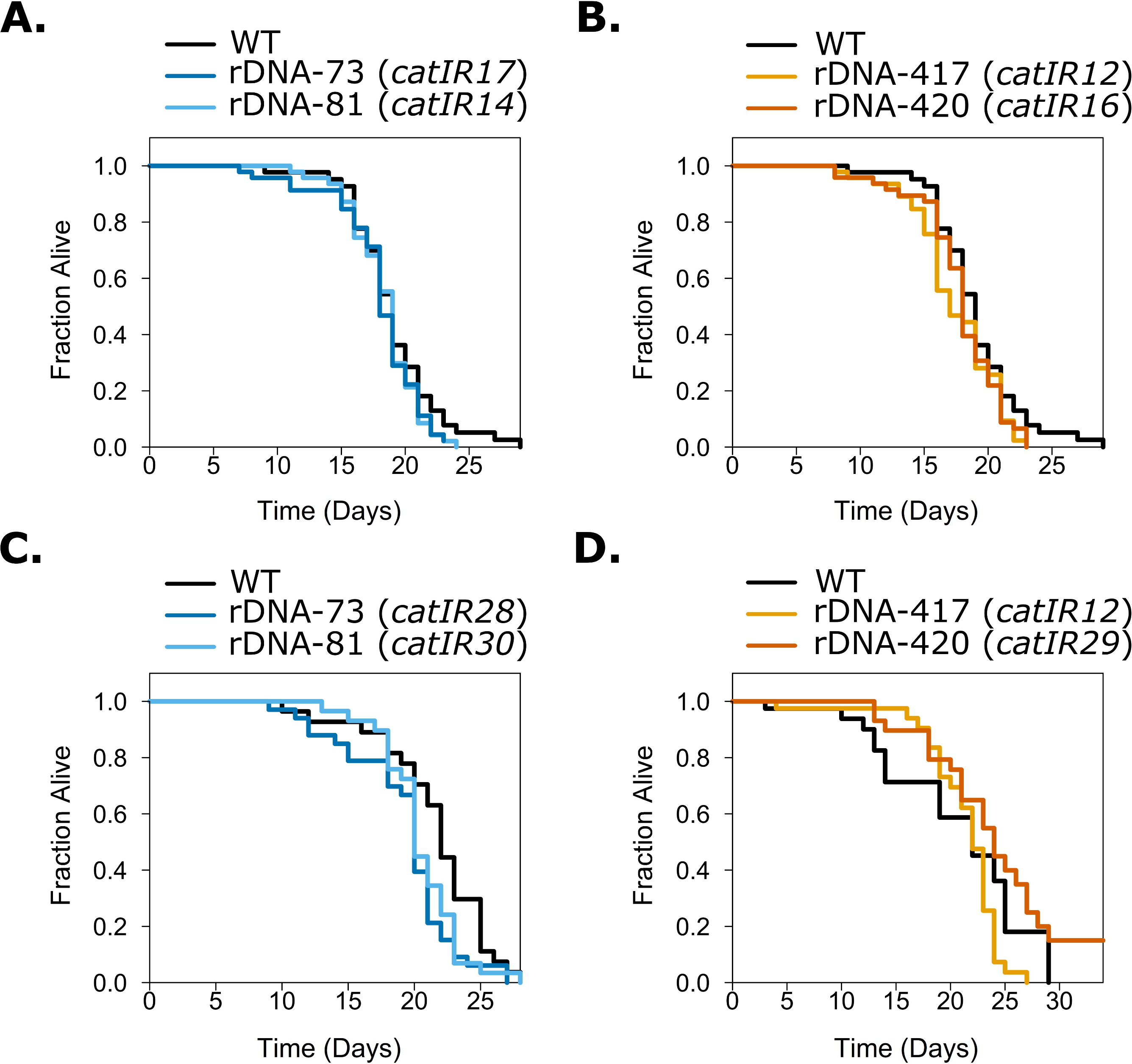

Supplement: S7 Fig — A and B: Lifespans of NILs with large linked wild isolate DNA regions (see Fig 2) were compared to N2. Lifespans for all five strains in A and B were performed simultaneously (panels A and B present the same N2 data)(n = 50). C and D: Lifespans of NILs with minimal linked wild isolate DNA (see Fig 2) were compared to N2 in biological replicates of the experiment presented in Fig 4. C: (n = 50). D: (n=40 (N2), n = 44 (rDNA-417), n = 42 (rDNA-420)). A significant difference (p = 0.01) was detected between N2 and rDNA-73 in the replicate presented in panel C only. (TIF) [file pgen.1011759.s007.tif]

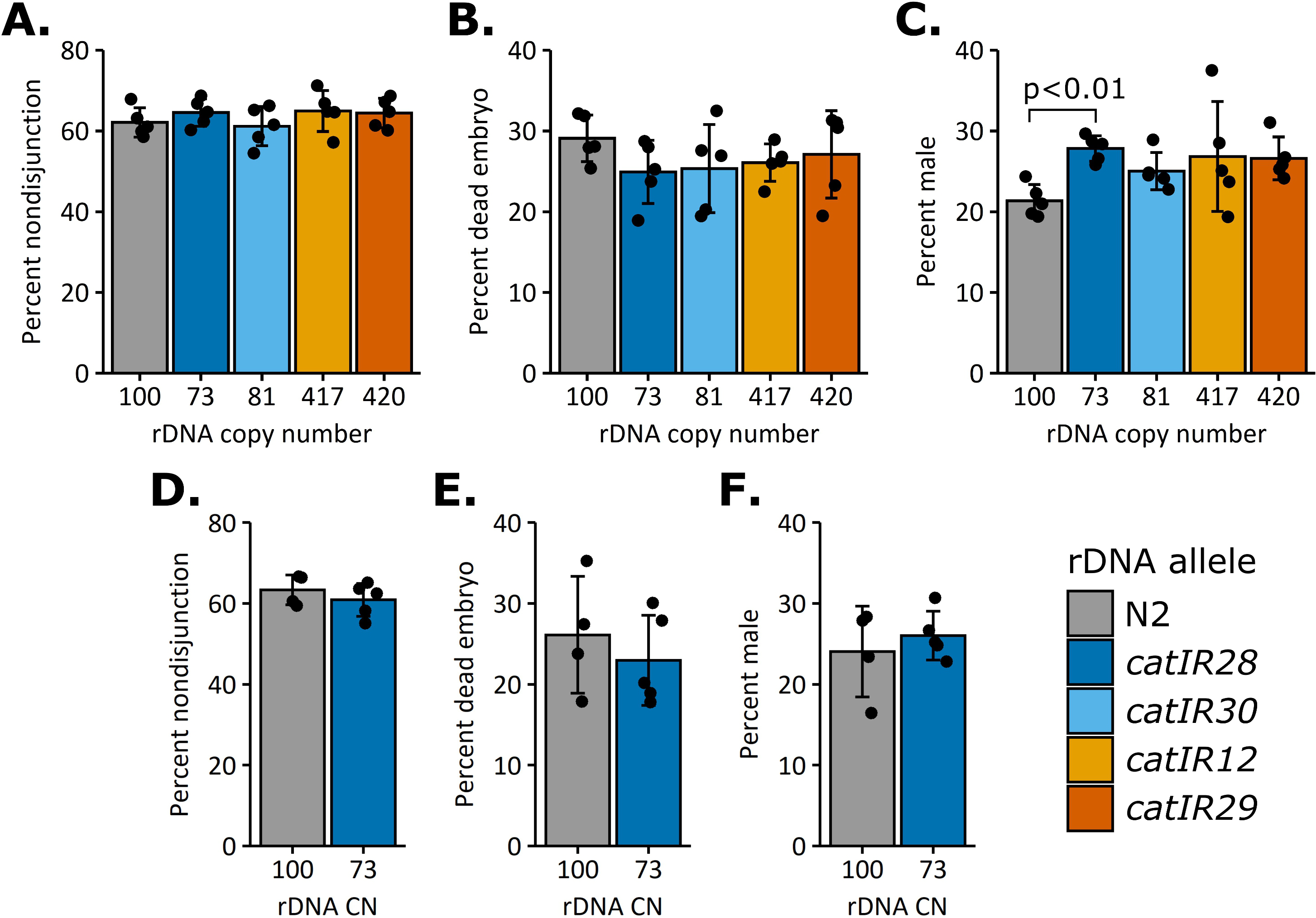

Supplement: S8 Fig — A: A mutation in a gene involved in meiotic break positioning, him-5(ok1986), was crossed into the rDNA copy number NILs to create strains sensitized for nondisjunction events. Progeny were then scored for incidence of males, dumpy worms, dead embryos, and other defects such as slow growth. The percent of progeny exhibiting such phenotypes is collectively plotted here as “nondisjunction”. Progeny from five adult worms were assessed per strain. No significant differences between any strains were observed by t-test and Bonferroni correction. B: The data from A are parsed out to present only the percent of progeny that failed to hatch (dead embryos) in NILs with him-5(ok1986). Dead embryos are assumed to arise from autosomal nondisjunction events. No significant differences were observed between any strains by t-test and Bonferroni correction. C: The data from A are parsed out to present only the percent male progeny (X-chromosome nondisjunction) in NILs with him-5(ok1986). The 73-rDNA NIL (allele catIR28) differed significantly from wild type in the percent male progeny, p < 0.01. D-F: A second replicate was conducted as in A-C, assessing only N2 and the 73-rDNA NIL. No significant differences were observed between these strains in collective nondisjunction events (D) or the individual contributions of dead embryos (E) or male frequency (F) (student’s t-test). (TIF) [file pgen.1011759.s008.tif]

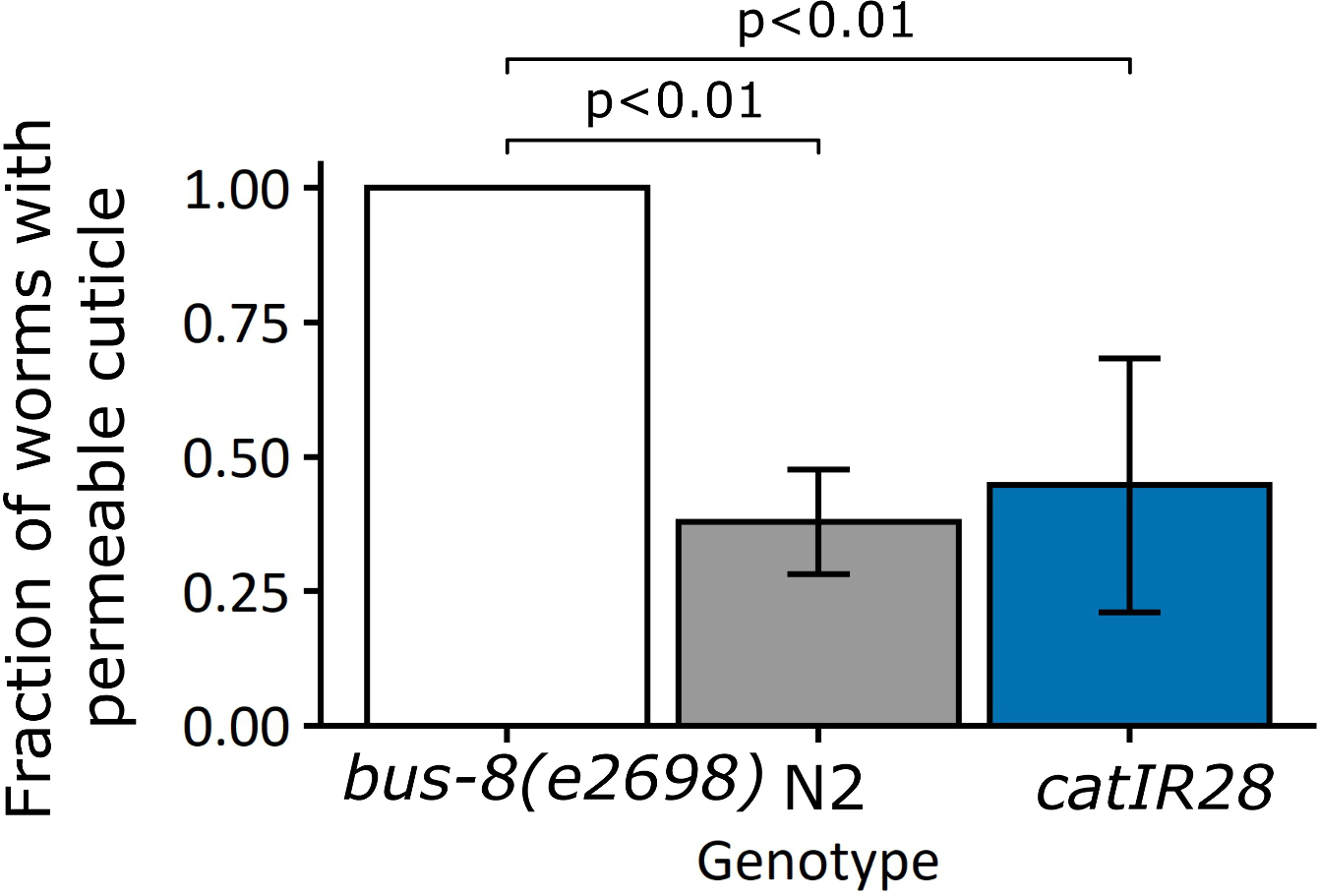

Supplement: S9 Fig — Worms were stained with Hoechst, which does not normally penetrate intact worm cuticles. Worms that exhibited staining in their hypodermal nuclei were scored as having permeable cuticles. At least 13 worms were quantified for each replicate, three replicates per genotype (S22 File). The bus-8(e2698) genotype was used as a positive control with known reduced cuticle integrity [139]; in all three replicates of this genotype all worms had permeable cuticles. N2 and the 73-rDNA NIL (catIR28) have a fraction of worms with permeable cuticles and do not differ from one another in cuticle permeability, as measured by ANOVA and Tukey’s HSD. Error bars represent standard deviation of the percent permeable over the three replicates. (TIF) [file pgen.1011759.s009.tif]
