# Supplementary material for: Phenotypic tolerance for rDNA copy number variation within the natural range of C. elegans
Source: PLoS Genet. 2025 Jul 2;21(7):e1011759. doi: 10.1371/journal.pgen.1011759 (PMC12221044; doi:10.1371/journal.pgen.1011759)
Supplement: S1 Table — (DOCX) [file pgen.1011759.s010.docx]

**Table S1: Comparison of rDNA copy number estimates of RILs from WGS and CHEF gel.**

| RIL ID | Strain name | WGS | CHEF |
| --- | --- | --- | --- |
| AG6 | SEA87 | 116.6 | 119 |
| BG4 | SEA113 | 97.5 | 90 |
| BG6 | SEA115 | 151.9 | 123.5* |
| BG8 | SEA117 | 134 | 118 |
| BG15 | SEA124 | 143 | 118 |
| AG22 | SEA102 | 127 | 119 |

*Average of two bands.
