# Supplementary material for: Phenotypic tolerance for rDNA copy number variation within the natural range of C. elegans
Source: PLoS Genet. 2025 Jul 2;21(7):e1011759. doi: 10.1371/journal.pgen.1011759 (PMC12221044; doi:10.1371/journal.pgen.1011759)
Supplement: S2 Table — (DOCX) [file pgen.1011759.s011.docx]

**Table S2: Gene Ontology Enrichment Analysis of genes differentially expressed in the 73-rDNA (allele *catIR28*) NIL as compared to N2.**

| **Term** | **Expected** | **Observed** | **Enrichment Fold Change** | **P value** | **Q value** |
| --- | --- | --- | --- | --- | --- |
| molting cycle GO:0042303 | 0.7 | 12 | 17 | 3.50E-13 | 1.00E-10 |
| structural constituent of cuticle GO:0042302 | 1.1 | 13 | 12 | 4.50E-12 | 6.60E-10 |
| collagen trimer GO:0005581 | 1.1 | 11 | 10 | 1.10E-09 | 1.00E-07 |
| cuticle development GO:0042335 | 0.36 | 7 | 19 | 3.10E-09 | 2.30E-07 |
| extracellular region GO:0005576 | 3.7 | 18 | 4.8 | 1.10E-08 | 6.70E-07 |
| Notch signaling pathway GO:0007219 | 0.32 | 4 | 13 | 1.70E-05 | 0.00084 |
| external encapsulating structure GO:0030312 | 0.54 | 5 | 9.3 | 1.80E-05 | 0.00084 |
| calcium ion binding GO:0005509 | 0.99 | 5 | 5.1 | 0.0005 | 0.018 |
| double-stranded DNA binding GO:0003690 | 2.8 | 9 | 3.2 | 0.00059 | 0.019 |
| protein-DNA complex organization GO:0071824 | 0.4 | 3 | 7.4 | 0.00074 | 0.022 |
| molecular function inhibitor activity GO:0140678 | 0.77 | 4 | 5.2 | 0.0011 | 0.029 |
| nucleosome GO:0000786 | 0.45 | 3 | 6.6 | 0.0011 | 0.029 |
| defense response to Gram-positive bacterium GO:0050830 | 0.47 | 3 | 6.4 | 0.0013 | 0.029 |
| structural constituent of cytoskeleton GO:0005200 | 0.22 | 2 | 9.1 | 0.0014 | 0.029 |
| DNA-binding transcription factor activity GO:0003700 | 3.8 | 10 | 2.6 | 0.0017 | 0.033 |
| neuron development GO:0048666 | 1.3 | 5 | 4 | 0.0018 | 0.033 |
| structural constituent of chromatin GO:0030527 | 0.51 | 3 | 5.8 | 0.0018 | 0.033 |
| dendritic tree GO:0097447 | 0.89 | 4 | 4.5 | 0.0021 | 0.035 |
| response to biotic stimulus GO:0009607 | 2.9 | 8 | 2.8 | 0.0028 | 0.043 |
| biological process involved in interspecies interaction between organisms GO:0044419 | 2.9 | 8 | 2.8 | 0.0028 | 0.043 |
| defense response GO:0006952 | 2.9 | 8 | 2.7 | 0.003 | 0.043 |
| transcription regulatory region nucleic acid binding GO:0001067 | 2.5 | 7 | 2.8 | 0.0039 | 0.052 |
| apical part of cell GO:0045177 | 0.66 | 3 | 4.6 | 0.0044 | 0.055 |
| cell projection organization GO:0030030 | 2.1 | 6 | 2.8 | 0.0058 | 0.071 |
| cellular developmental process GO:0048869 | 5.8 | 12 | 2.1 | 0.0062 | 0.072 |
| immune system process GO:0002376 | 2.3 | 6 | 2.7 | 0.008 | 0.09 |
