# Supplementary material for: Phenotypic tolerance for rDNA copy number variation within the natural range of C. elegans
Source: PLoS Genet. 2025 Jul 2;21(7):e1011759. doi: 10.1371/journal.pgen.1011759 (PMC12221044; doi:10.1371/journal.pgen.1011759)
Supplement: S3 Table — (DOCX) [file pgen.1011759.s012.docx]

**Table S3: Phenotype Enrichment Analysis of genes differentially expressed in the 73-rDNA (allele *catIR28*) NIL as compared to N2**

| **Term** | **Expected** | **Observed** | **Enrichment Fold Change** | **P value** | **Q value** |
| --- | --- | --- | --- | --- | --- |
| paralyzed WBPhenotype:0000644 | 1.2 | 9 | 7.7 | 3.40E-07 | 8.00E-05 |
| dumpy WBPhenotype:0000583 | 2.5 | 13 | 5.1 | 3.70E-07 | 8.00E-05 |
| pericellular component development variant WBPhenotype:0000200 | 0.68 | 7 | 10 | 4.90E-07 | 8.00E-05 |
| molt variant WBPhenotype:0002041 | 1.8 | 9 | 5 | 1.60E-05 | 0.00096 |
| movement variant WBPhenotype:0001206 | 13 | 26 | 2 | 0.00026 | 0.012 |
| age associated fluorescence increased WBPhenotype:0000467 | 0.57 | 4 | 7 | 0.00029 | 0.012 |
| breaks in alae WBPhenotype:0000280 | 0.32 | 3 | 9.3 | 0.00031 | 0.012 |
| stress induced lethality variant WBPhenotype:0000139 | 0.42 | 3 | 7.2 | 0.00083 | 0.025 |
| intestinal vacuole WBPhenotype:0001428 | 0.42 | 3 | 7.2 | 0.00083 | 0.025 |
| protein degradation variant WBPhenotype:0001645 | 1.2 | 5 | 4.3 | 0.0012 | 0.028 |
| male mating efficiency reduced WBPhenotype:0000843 | 0.46 | 3 | 6.5 | 0.0012 | 0.028 |
| body morphology variant WBPhenotype:0000072 | 8.9 | 18 | 2 | 0.0018 | 0.037 |
| body region phenotype WBPhenotype:0002557 | 9.9 | 19 | 1.9 | 0.0027 | 0.05 |
| antihelmintic response variant WBPhenotype:0001852 | 0.32 | 2 | 6.2 | 0.0042 | 0.071 |
| organism hypertonic lethality increased WBPhenotype:0001751 | 0.33 | 2 | 6.1 | 0.0044 | 0.071 |
