# Supplementary material for: Phenotypic tolerance for rDNA copy number variation within the natural range of C. elegans
Source: PLoS Genet. 2025 Jul 2;21(7):e1011759. doi: 10.1371/journal.pgen.1011759 (PMC12221044; doi:10.1371/journal.pgen.1011759)
Supplement: S4 Table — (DOCX) [file pgen.1011759.s013.docx]

**Table S4: Tissue Enrichment Analysis of genes differentially expressed in the 73-rDNA (allele *catIR28*) NIL as compared to N2**

| **Term** | **Expected** | **Observed** | **Enrichment Fold Change** | **P value** | **Q value** |
| --- | --- | --- | --- | --- | --- |
| epithelial system WBbt:0005730 | 28 | 72 | 2.6 | 7.70E-14 | 2.30E-11 |
| excretory duct cell WBbt:0004540 | 0.25 | 5 | 20 | 2.00E-07 | 3.00E-05 |
| outer labial sensillum WBbt:0005501 | 16 | 38 | 2.4 | 3.40E-07 | 3.40E-05 |
| PVD WBbt:0006831 | 15 | 37 | 2.4 | 5.80E-07 | 4.30E-05 |
| hyp6 WBbt:0004679 | 0.31 | 4 | 13 | 1.70E-05 | 0.001 |
| touch receptor neuron WBbt:0005237 | 11 | 22 | 2.1 | 0.00051 | 0.026 |
| HSN WBbt:0006830 | 0.69 | 4 | 5.8 | 0.00071 | 0.03 |
| P5 WBbt:0006774 | 0.18 | 2 | 11 | 0.00073 | 0.03 |
| P8 WBbt:0006777 | 0.18 | 2 | 11 | 0.00073 | 0.03 |
| P1 WBbt:0006770 | 0.18 | 2 | 11 | 0.00073 | 0.03 |
| P2 WBbt:0006771 | 0.18 | 2 | 11 | 0.0008 | 0.03 |
| P6 WBbt:0006775 | 0.18 | 2 | 11 | 0.0008 | 0.03 |
| P9 WBbt:0006778 | 0.19 | 2 | 11 | 0.00087 | 0.03 |
| P7 WBbt:0006776 | 0.19 | 2 | 11 | 0.00087 | 0.03 |
| P4 WBbt:0006773 | 0.19 | 2 | 11 | 0.00087 | 0.03 |
| P10 WBbt:0006779 | 0.21 | 2 | 9.4 | 0.0013 | 0.03 |
| P12 WBbt:0004409 | 0.26 | 2 | 7.8 | 0.0022 | 0.039 |
| hyp5 WBbt:0004685 | 0.28 | 2 | 7.2 | 0.0028 | 0.046 |
| hyp4 WBbt:0004687 | 0.29 | 2 | 6.8 | 0.0032 | 0.051 |
| P11 WBbt:0004410 | 0.31 | 2 | 6.5 | 0.0038 | 0.056 |
| male WBbt:0007850 | 14 | 24 | 1.7 | 0.0046 | 0.066 |
| PVQ WBbt:0006976 | 0.34 | 2 | 5.9 | 0.005 | 0.068 |

For the P1-P12 tissue enrichments that show up in the TEA, the two genes that underlie the enrichment are the same for each: *hbl-1* and *dpy-7.* These P1-P12 terms are a set of twelve postembryonic blast cells.
