# Supplementary material for: Phenotypic tolerance for rDNA copy number variation within the natural range of C. elegans
Source: PLoS Genet. 2025 Jul 2;21(7):e1011759. doi: 10.1371/journal.pgen.1011759 (PMC12221044; doi:10.1371/journal.pgen.1011759)
Supplement: S5 Table — (DOCX) [file pgen.1011759.s014.docx]

**Table S5: Strains used in this study**

| Strain | Genotype | Source |
| --- | --- | --- |
| VC2010 | N2 (WT) | Waterston lab |
| MY1 | Wild isolate MY1 | Moerman lab |
| JU775 | Wild isolate JU775 | Moerman lab |
| MY16 | Wild isolate MY16 | Moerman lab |
| RC301 | Wild isolate RC301 | Teotonio lab |
| CB1370 | *daf-2*(*e1370*) III | CGC |
| PS3551 | *hsf-1*(*sy441*) I | CGC |
| SEA51 | *mIs13*[myo-2p::GFP + pes-10p::GFP + F22B7.9p::GFP] I | This study |
| SEA300 | *catIR12* [MY1 chrI:13527418-end] | This study |
| SEA302 | *catIR14* [JU775 I:13529383-end] | This study |
| SEA304 | *catIR16* [RC301 I:11737213-end] | This study |
| SEA305 | *catIR17* [MY16 I:3576802-end] | This study |
| SEA328 | *catIR28* I [MY16 I:14764185-end] | This study |
| SEA329 | *catIR29* I [RC301 I:14989978-end] | This study |
| SEA330 | *catIR30* I [JU775 I:14775350-end] | This study |
| RB1562 | D1086.4(*ok1896*) V | CGC |
| SEA333 | D1086.4(*ok1896*) V | This study |
| SEA340 | *catIR12 him-5*(*ok1896*) | This study |
| SEA344 | *catIR28 him-5*(*ok1896*) | This study |
| SEA345 | *catIR29 him-5*(*ok1896*) | This study |
| SEA346 | *catIR30 him-5*(*ok1896*) | This study |

*RIL strains are listed in a separate table: See Supplemental File S1.
