# Supplementary material for: Phenotypic tolerance for rDNA copy number variation within the natural range of C. elegans
Source: PLoS Genet. 2025 Jul 2;21(7):e1011759. doi: 10.1371/journal.pgen.1011759 (PMC12221044; doi:10.1371/journal.pgen.1011759)
Supplement: S6 Table — (DOCX) [file pgen.1011759.s015.docx]

**Table S6: Genotyping loci for NIL generation**

| Marker | Description | Genotyping usage |
| --- | --- | --- |
| *mIs13* | GFP (predominantly pharynx) presence vs absence | SEA51 (laboratory background rDNA copy number) has *mIs13* genetically linked to rDNA locus |
| 12.55 Mb | *HhaI* RFLP amplified by AHC13+AHC14 | RC301 and MY16 wild isolate sequence cuts with HhaI; N2 does not |
| 14.5 Mb | Deletion amplified with primers AHC1+AHC2 | JU775 wild isolate has 300bp deletion as compared to N2 |
| 14.68 Mb | SNV detected with genotyping primers | AHC37+AHC48 amplify N2; AHC37+AHC49 amplify MY1, RC301, JU775, and MY16 |
| 14.99 Mb | *MnlI* RFLP amplified with primers AGC32+AHC35 | MY1, RC301, JU775, and MY16 wild isolate sequence do not cut; N2 cuts. This genotyping locus is retained in the NILs (SEA328, SEA329, SEA330, SEA300) |
