# Supplementary material for: Phenotypic tolerance for rDNA copy number variation within the natural range of C. elegans
Source: PLoS Genet. 2025 Jul 2;21(7):e1011759. doi: 10.1371/journal.pgen.1011759 (PMC12221044; doi:10.1371/journal.pgen.1011759)
Supplement: S7 Table — (DOCX) [file pgen.1011759.s016.docx]

**Table S7: Primers used in this study**

| Primer name | Primer sequence | Description | Source |
| --- | --- | --- | --- |
| AHC1 | GGTCAAAATCGAAAGTGGAAAC | ChrI 14.5 Mb F | This study |
| AHC2 | GTGAAAGTGTAGAAAAAGTCTTTAGAAATAG | ChrI 14.5 Mb R | This study |
| AHC14 | GACAAAACATGATCAAATGAGAAC | ChrI 12.5 Mb R | This study |
| AHC24 | GCACCATTTGGAGCTATG | ChrI 12.5 Mb F | This study |
| AHC32 | CCATTTTTGCACCATTGGAG | ChrI 14.99 Mb F | This study |
| AHC35 | CAAGCGCCTTTATTGAAAAGC | ChrI 14.99 Mb R | This study |
| AHC36 | GTACCTATTTCTTCCCACCTG | ChrI 14.68 Mb F | This study |
| AHC55 | ACCGACGGTAACTAAGATTT | ChrI 14.68 Mb R1 | This study |
| AHC56 | CGACGGTAACTAAGATCC | ChrI 14.68 Mb R2 | This study |
| AHC66 | GTGTCCCATCTCACGATTAG | Probe I F | (Wu *et al*. 2018) |
| AHC67 | GTGATATCTGCTCTAATGAG | Probe I R | (Wu *et al*. 2018) |
| AHC68 | AACGACTTCGTTGTTGCGG | Probe II F | (Wu *et al*. 2018) |
| AHC69 | TTCGACACTCAACTGACCG | Probe II R | (Wu *et al*. 2018) |
| AHC70 | TCAACGTTCCAGTTGAGATG | Probe III F | (Wu *et al*. 2018) |
| AHC71 | CGATCATCAAGACTATCGTC | Probe III R | (Wu *et al*. 2018) |
| AHC72 | TGGCTATATGCGTCTAGGC | Probe IV F | (Wu *et al*. 2018) |
| AHC73 | ATCACCGCATGTCCGTGAAG | Probe IV R | (Wu *et al*. 2018) |
| AHC74 | CTTCACGGACATGCGGTGAT | Probe V F | (Wu *et al*. 2018) |
| AHC75 | AGTTGGTGCTATGCGTTCG | Probe V R | (Wu *et al*. 2018) |
| AHC76 | CGAACGCATAGCACCAACT | Probe VI F | (Wu *et al*. 2018) |
| AHC77 | TGTGATGCTTCTGGACTAGG | Probe VI R | (Wu *et al*. 2018) |
| AHC78 | TCGAATACTGGGATTCGTC | Probe VII F | (Wu *et al*. 2018) |
| AHC79 | AGCAGCCAAAGACTGATCG | Probe VII R | (Wu *et al*. 2018) |
| AHC84 | TCGGTATGGGACAGAAGGAC | Actin F | (Lee *et al*. 2014) |
| AHC85 | CATCCCAGTTGGTGACGATA | Actin R | (Lee *et al*. 2014) |
| AHC88 | GCTTACGACCATATCACGTTGAATG | 5S_qPCR_F | This study |
| AHC89 | CTTACAACATCCAGGATTCCCAG | 5S_qPCR_R | This study |
| EM50 | CGAGGTCTCCAGAGAGACG | Southern rDNA probe | (Morton *et al*. 2020) |
| EM51 | AGTTGAAAGGGCAGACACCC | Southern rDNA probe | (Morton *et al*. 2020) |
